# Thermotaxis in an apolar, non-neuronal animal

**DOI:** 10.1098/rsif.2023.0279

**Published:** 2023-09-13

**Authors:** Grace Zhong, Laurel Kroo, Manu Prakash

**Affiliations:** ^1^ Department of Bioengineering, Stanford University, Stanford, CA 94305, USA; ^2^ Department of Mechanical engineering, Stanford University, Stanford, CA 94305, USA; ^3^ Woods Institute for the Environment, Stanford University, Stanford, CA 94305, USA

**Keywords:** *Trichoplax*, apolar, thermotaxis, animal behaviour, non-neuronal

## Abstract

Neuronal circuits are hallmarks of complex decision-making processes in the animal world. How animals without neurons process information and respond to environmental cues promises a new window into studying precursors of neuronal control and origin of the nervous system as we know it today. Robust decision making in animals, such as in chemotaxis or thermotaxis, often requires internal symmetry breaking (such as anterior–posterior (AP) axis) provided naturally by a given body plan of an animal. Here we report the discovery of robust thermotaxis behaviour in *Trichoplax adhaerens*, an early-divergent, enigmatic animal with no anterior–posterior symmetry breaking (apolar) and no known neurons or muscles. We present a quantitative and robust behavioural response assay in *Placozoa*, which presents an apolar flat geometry. By exposing *T. adhaerens* to a thermal gradient under a long-term imaging set-up, we observe robust thermotaxis that occurs over timescale of hours, independent of any circadian rhythms. We quantify that *T. adhaerens* can detect thermal gradients of at least 0.1°C cm^−1^. Positive thermotaxis is observed for a range of baseline temperatures from 17°C to 22.5°C, and distributions of momentary speeds for both thermotaxis and control conditions are well described by single exponential fits. Interestingly, the organism does not maintain a fixed orientation while performing thermotaxis. Using natural diversity in size of adult organisms (100 µm to a few millimetres), we find no apparent size-dependence in thermotaxis behaviour across an order of magnitude of organism size. Several transient receptor potential (TRP) family homologues have been previously reported to be conserved in metazoans, including in *T. adhaerens*. We discover naringenin, a known TRPM3 antagonist, inhibits thermotaxis in *T. adhaerens*. The discovery of robust thermotaxis in *T. adhaerens* provides a tractable handle to interrogate information processing in a brainless animal. Understanding how divergent marine animals process thermal cues is also critical due to rapid temperature rise in our oceans.

## 1. Introduction

It has been said that ‘nothing in neuroscience makes sense except in the light of behaviour’ [1] or ‘evolution’ [2]. How organisms integrate and transform sensory information into behavioural responses has both fascinated and confounded us since the days of Aristotle. Most behavioural responses in traditional biological model systems involve complex neuronal circuits [3]. In these systems, complexity arises at emergent length scales—captured by the expression ‘more is different’ [4]. Reductionist approaches often fail to capture these systems completely [5].

Historically, ‘simpler’ systems have been remarkably useful to identify key motifs in origins of behaviour [6] (which may be translatable to other organisms). A large number of such systems with fewer neurons (e.g. *Caenorhabditis elegans*, *Drosophila* larvae, lobster stomach) have provided hallmark studies enabling reduction of complex behaviours into sensory-motor transformations [3,7,8]. However, even in these simpler model systems, it is still difficult to elucidate functional mapping at whole organism scale [9]. In addition, biological systems usually demonstrate stereotypical architectures, and it is difficult for an experimentalist to ‘scale the size’ of the system. As an extreme example, *C. elegans* always contains 302 neurons [9], and in less extreme examples, it is still difficult to decouple size of the organism from factors such as age. Thus, a comparative approach (so common in physics) is often difficult to implement. These challenges inspire us to study behavioural diversity in a broader range of non-model systems across the animal tree of life—with a possibility to circumnavigate these challenges. Since the explosion of multicellularity preceded the emergence of nervous systems, an illuminating question that arises is, how do multicellular animals without neurons or muscles, and without ‘nervous systems’ in the current-day definition, perceive and respond to environmental stimuli and make decisions? What are the limits of information processing capacity in these systems, and how do the limits map to the size of the organism? In our current work, we explore this space of sensory-motor transformation at the ‘zero-brain limit’.

In the context of animal behaviour, generation of a directed migration in response to a stimulus is typically reported in systems that are polarized [10,11]. For most animals, this polarization/symmetry breaking is inherent in the body plan through anterior–posterior (AP) symmetry breaking [12]. Even single cells such as neutrophils rapidly adopt a polarized morphology when presented with a chemical gradient [13]. From single cells to cellular collectives, it is generally accepted that the direction of migration is governed by leading cells. For example, in the invasion of carcinoma cells, the leading cells are always fibroblasts [14]. Some examples highlight the role of leader cell turnover [15], such as in chemotaxing lymphoid cell clusters [16]. Here we intend to explore if it is possible for an apolar animal without inherent AP symmetry breaking to make persistent long-term decisions, such as in climbing up a shallow thermal gradient. What would govern the fundamental limits of sensitivity [17] in such a system? How would these limits change depending on the size of the organism and total number of cells engaged in the process?

To better elucidate the role of size and symmetry breaking in taxis behaviour, we seek to use a simple environmental cue as a handle. Temperature is a physiologically critical parameter that is crucial for survival of all life forms [18]. Thus temperature gradients are important ubiquitous environmental stimuli that influence behaviour [6] in both terrestrial and marine ecosystems. Furthermore, an urgency exists in understanding how marine animals (both adult and larval forms) process and adapt to thermal cues in face of rising ocean temperatures. Examples include settlement behaviour of starfish [19] and coral larvae [20], adaptation strategies of annelids in extreme temperatures [21], and worms that thrive near hydrothermal vents [22].

Owing to their broad applicability, thermal sensing capabilities and thermotaxis behaviour have been reported ubiquitously in unicellular and multicellular systems. Most animals have been reported to be temperature-sensitive with a sensitivity of the order of 0.1°C cm^−1^. Examples include humans (0.1°C) and human sperm (0.14°C cm^−1^) [23,24], *C. elegans* (0.1°C) [25] and *Drosophila* larvae (0.23°C cm^−1^) [7]. As an example of an extreme temperature-sensitivity, the nematode *Meloidogyne incognita* has a reported sensitivity limit below 0.001°C cm^−1^ [26].

This example pushes the fundamental limits of what might be possible to build using molecular components in a thermally noisy environment. Focusing specifically on non-neuronal systems, thermotaxis behaviour has been reported both in organisms that transiently form multicellular structures (*Dictyostelium discoideum* [27]) and in single-celled organisms (phytoplanktonic flagellates [28] and *E. coli* [29]).

There has also been extensive work in characterizing and elucidating the mechanisms behind thermotaxis strategies of various organisms. To give some examples (by no means an extensive sampling of the amazing bodies of work that has been done), *E. coli* tend to perform positive thermotaxis at low temperatures and negative thermotaxis at high temperatures, resulting in accumulation at intermediate temperatures through control of tumble frequency [30,31], and aggregation temperature might depend on gradient steepness [30]. *Caenorhabditis elegans* senses temperature using AFD neurons; it also demonstrates bi-directional thermotaxis and shows preference for culture temperature, tracking isotherms with 0.1°C precision [25,32]. Its run length distributions are exponentially distributed [32], and it generally uses the strategy of biased random walk in navigating up and down gradients [25]. *Drosophila* larvae also show bi-directional thermotaxis, biasing run duration and direction of turning decisions [33].

**Figure 1.**
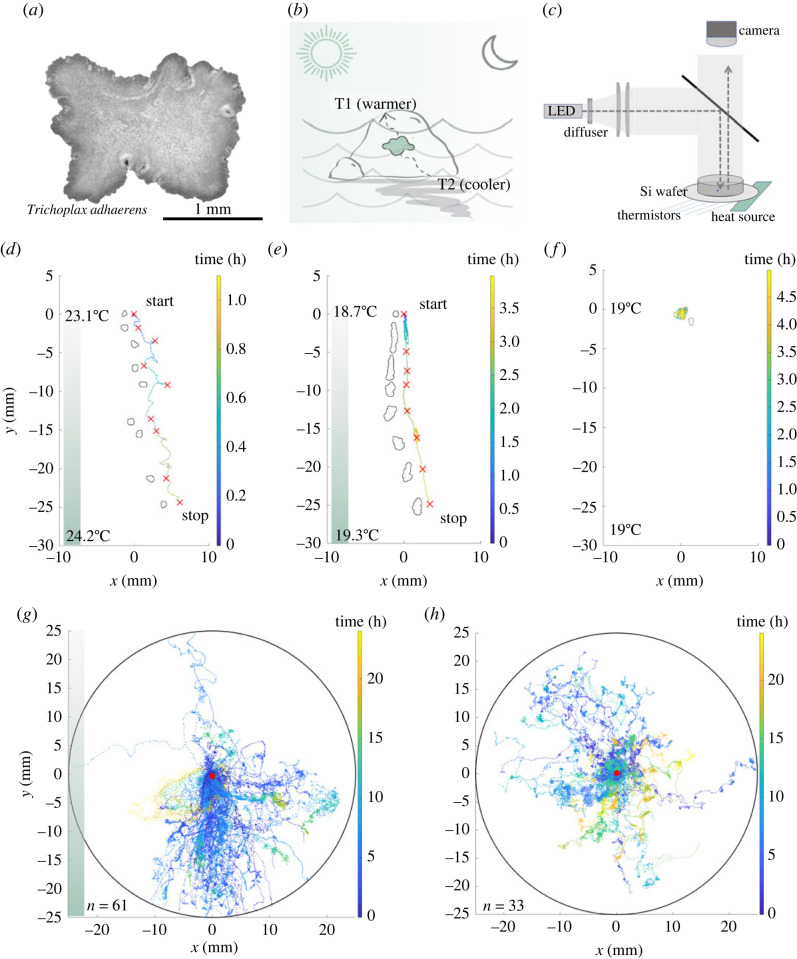
Discovery of robust thermotaxis in *T. adhaerens*—an organism in the phylum *Placozoa.* (*a*) *Trichoplax adhaerens* has an apolar, flat geometry. (*b*) Thermal cues play an important role in their shallow-water natural environment. Temperatures would vary depending on factors like tides, seasons, time of day and shadows. (*c*) Experiment set-up using flexible heaters and overhead camera. The heating elements are attached to the bottom of the Si wafer, but not directly under the arena area delineated by the PVC chamber. (*d*,*e*) Sample organism trajectories show directed motion of organism towards higher temperatures under two different thermal gradients. Colour bar indicates time. Segmented shape of the organism is shown at various time points. As is typical of *T. adhaerens* motile trajectories, the organism changes shape and projected two-dimensional area throughout the trajectory (see electronic supplementary material, figure S2 for corresponding temperature profiles). (*f*) A sample control trajectory with no thermal gradient applied shows no directed motion. (*g*,*h*) Overlaid trajectories of biologically independent trials for heat (*g*) (*n* = 61) and control (*h*) (*n* = 33) conditions. T1 (temperature at warmer side of arena) range for trials shown is 17.3–28.2°C; T2 (temperature at colder side of arena) range is 13.3–26.7°C; Δ*T* range is 0.008–0.42°C cm^−1^.

Here we explore what strategies apolar, non-neuronal animals use to navigate their environments. To focus on this question, here we study the response of *Trichoplax adhaerens* to thermal gradients. *Trichoplax adhaerens* (figure 1*a*) belongs to phylum *Placozoa* and is the only known metazoan with no known AP symmetry axis. The organism is composed of two epithelial layers in a flattened geometry—akin to a deflated beach ball. Furthermore, the animal does not have known neurons or muscles—it propels itself in an unusual fashion, coordinating millions of ‘walking cilia’ [34] with asynchronous beats. These resulting ‘ciliary flocking’ dynamics are rich yet discernible [34–36], providing a mechanical analogue of behavioural control in these animals [36]. Since both microscale dynamics and whole animal behaviour can be studied in controlled conditions in the laboratory, *T. adhaerens* is a remarkable and experimentally tractable system to explore origins of information processing in non-neuronal animals. In this system, with millions of individual ‘walking cilia’ with excitable dynamics, we can find analogies to principles commonly explored in neuronal ensembles. Specifically, does stimulus cause behavioural response in a ‘passive nervous system’, or does stimulus modulate ongoing behaviour generated by an ‘active nervous system’? [37] Here, the debate between ‘motor-sensory’ and ‘sensory-motor’ models extends beautifully to the non-neuronal, mechanical analogue present in the walking cilia of *T. adhaerens*.

Ecologically, *T. adhaerens* has been previously collected in tropical shallow waters around the globe, crawling on surfaces covered with algae mats. Although their behaviour has never been observed *in situ*, large temperature fluctuations are common in shallow waters due to factors such as time of day, rock coverage and seasonal changes in water temperature (figure 1*b*). Thus, being able to respond to temperature fluctuations can play an important role in the survival of any benthic species. Although the complete life cycle of any reported species in the phylum *Placozoa* (including *T. adhaerens*) has not been recapitulated in the laboratory, adult animal cultures can be grown at scale in laboratory conditions. This allows us to address behavioural questions in the context of this apolar, aneural system. The animal further shows a natural yet large variation in size, allowing us to ask a fundamental question of how animal behaviour varies with animal size. Here we explore the ‘zero-brain limit’ in animal behaviour—a condition that can be reasonably linked to precursors of early animal forms.

Herein we report the discovery of robust thermotaxis in *T. adhaerens*. Positive thermotaxis is robustly observed across the temperature range explored. Intriguingly, thermotaxis behaviour is observed over a period of several hours while the organism does not maintain a constant orientation in the frame of reference to the animal body. Characterizing the trajectories using quantitative behavioural analysis approaches enables us to elucidate relevant timescales for integration of gradient information. We also find that momentary displacements for both thermal gradient and control conditions can be well described by exponential functions. Further, we quantify the sensitivity of *T. adhaerens*'s ability to detect spatial thermal gradients to be least 0.1°C cm^−1^. We further explore whether organism size affects thermotaxis behaviour across an order of magnitude of organism sizes, and we find no apparent dependence of thermotaxis on organism size. Finally, we lay the groundwork for identifying molecular underpinnings of this behaviour by demonstrating reversible inhibition of thermotaxis by naringenin (a known antagonist of mouse TRPM3), suggesting the possibility that transient receptor potential (TRP) channel homologues known to exist in *T. adhaerens* may be involved. Thus, our work establishes a link between thermosensory circuitry and ‘ciliary flocking’.

## 2. Methods

### 2.1. Culture of *Trichoplax adhaerens*

Our cultures of *T. adhaerens* are propagated from original cultures that were a gift from L. Buss, Yale University. They are maintained in artificial seawater (ASW) with 3% salinity, in glass petri dishes. Cultures are kept on shelves in a dedicated room with temperature maintained at 18°C and light cycle of 18 h light–6 h dark.

ASW is prepared to reach 3% salinity by adding Kent Marine Reef Salt Mix or CoralPro Salt Mix in Millipore water on a magnetic stirrer, and salt concentration is measured by automatic temperature compensation (ATC) salinity refractometer.

The food source for the *T. adhaerens* culture is marine alga *Rhodomonas lens*, propagated from cultures that were gifted by C. Lowe lab (Stanford). *Rhodomonas lens* cultures are maintained in 500 ml sterile Erlenmeyer flasks either in dedicated incubators maintained at 14°C or in the same room as *T. adhaerens* (maintained at 18°C) on separate shelves. Micro Algae Grow from Florida Aqua Farms is used as a source of nutrients for *R. lens* cultures. Algae flasks are shaken daily.

For our *T. adhaerens* cultures, we start by adding 250 µl of filter-sterilized Micro Algae Grow per 1000 ml of filtered ASW to create an ASW stock. Then we fill the glass petri dish with 110 ml of the ASW stock and 15 ml of *R. lens*. We let the plate rest on the shelf for 2–3 days to allow for a growth of an ‘algal mat’ at the dish bottom, then we add 10–20 *T. adhaerens* to the plate.

We do a 30 ml water refresh once or twice weekly, and add 5 ml of algae during plate refreshes if the organisms no longer look pink in colour.

### 2.2. Behavioural assays

*Trichoplax adhaerens* were placed in nutrient-free ASW for 2–8 h prior to behavioural experiments for them to shed their undigested algae coat so as to remove chemical information from algae to isolate the effect of thermal information. They are then pipetted into the middle of the arena, which is filled with 25 ml of nutrient-free ASW, the heat source is turned on, and the acquisition (using custom Python script), is started.

The chamber for all described behavioural experiments except for the orientation experiment is made from an approximately 3 cm tall section of clear PVC piping with 5.25 cm inner diameter (McMaster Carr) attached to a silicon wafer (University Wafers) with silicone sealant. To the back of the wafer is attached, with thermally conductive tape, either a flexible polyimide heating strip (Omega) or two 11 W 5 V peltier modules (Laird Electronics DA-011-05-02). The chambers with flexible polyimide heating strip as the heating element are controlled by GW INSTEK GPD3303D benchtop power supply. The chambers with peltier modules as the heating/cooling elements are controlled by Wavelength Electronics WTCP5V5A PWM Temperature Controller mounted on evaluation boards from the same company. The lighting set-up is built with Thorlabs parts, and the image acquisition is done with Basler cameras (Basler acA2040-90um) attached to with Edmund Industrial Optics 35 mm Double Gauss Lens (Part number 54689). For chambers with thermistors, 10 kΩ thermistors are attached to the bottom of wafers using TG-PP10-50 thermal putty, then covered by tape. We measured the size of the organism by imaging under a higher magnification microscope prior to the beginning of the trials. Note that we have size information for 43 out of 61 trials because there was a period of time when the microscopes available for the size measurements were not functional and/or accessible. For three of the trials, we tried a chamber made using a larger silicon wafer (300 mm diameter from University Wafers). These trials are indicated as ‘large dish’ in the spreadsheet titled ‘Experiment_List.xlsx’ in our repository. Mean and standard deviations for total recording lengths are 12 h and 6 h, respectively, for control trials, and 7 h and 5 h, respectively, for thermal gradient trials (electronic supplementary material, figure S3).

A difficulty we experienced while probing the effect of size is that it is difficult to obtain trials with large organisms because they have a higher chance of sticking to the pipette (causing rupture), it is difficult to ensure that they have shed all algae (which would be a confounding signal in the dish) without starving them for so long that they almost certainly rupture during transfer, and they also have a higher chance of splitting during the trial.

**Figure 2.**
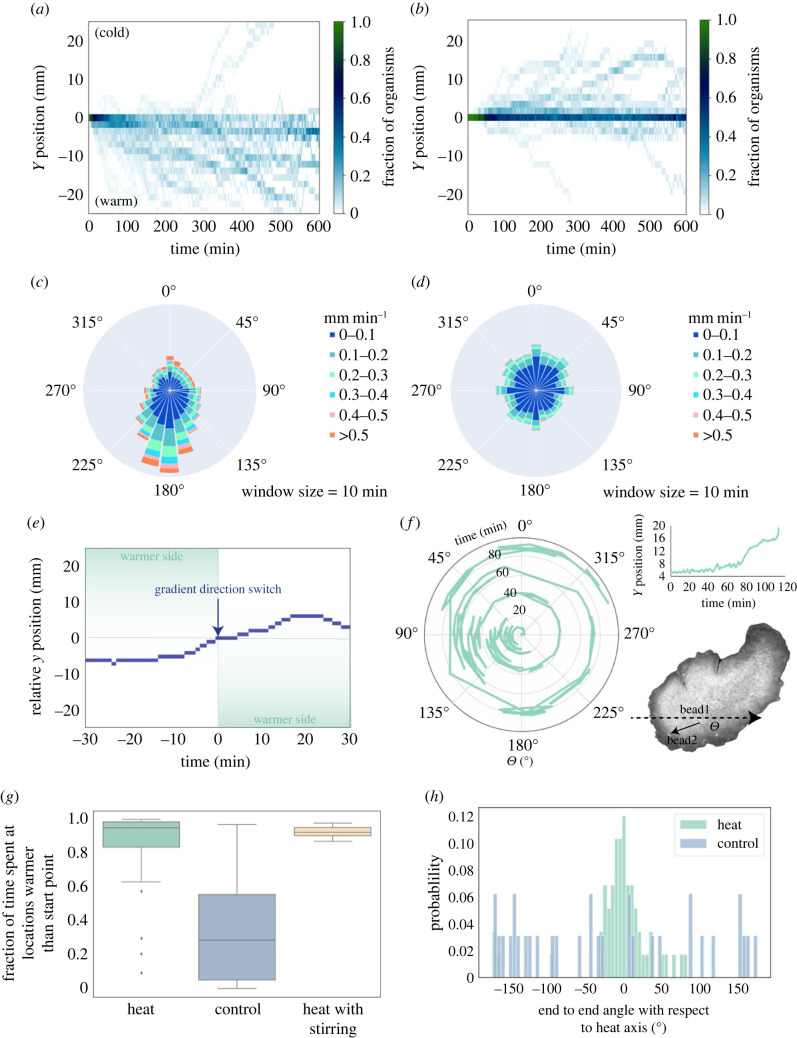
(*a,b*) Kymographs for heat (*a*) and control (*b*) conditions. When thermal gradient is applied, most organisms find the gradient within 10 h. Without a thermal gradient, organisms still sometimes show directed motion, but the direction is often random, and it appears that more organisms tarry near the start point for longer before exploring. (*c,d*) Windrose velocity diagrams for heat (*c*) and control (*d*) conditions using overlapping window size of 10 min. When thermal gradient is applied, motion is directed towards the heat, and velocities parallel to the heat axis are also higher. Bars are grouped by velocity range and bar height normalized to total number of points. Radial axis limit is 0.1. (*e*) It takes approximately 20 min for the organism to switch directions when the direction of the thermal gradient is instantaneously flipped while the organism is already moving towards the warmer side (see electronic supplementary material, video S4). (*f*) During thermotaxis, *T. adhaerens* does not have a fixed orientation (see electronic supplementary material, video S7). (Orientation is quantified as angle between ‘body axis’ and positive *x*-axis. ‘Body axis’ is defined using positions of two polystyrene beads attached to organism's dorsal surface.) (*g*) When a thermal gradient is present, the organism spends more time exploring locations that are warmer than the starting position. This holds even when an external water flow is generated in addition to presence of thermal gradient. (*h*) Distribution of end-to-end angle of trajectory (with respect to thermal gradient axis) for experiments with and without thermal gradient. When gradient is applied, end-to-end trajectories are highly aligned with the thermal axis. The distribution for trials with thermal gradient has a mean of 0.87° and standard deviation of 50°.

Behavioural data were pre-processed to get a trajectory using a custom MATLAB script where the organism's position is determined at each frame by thresholding and size-filtering until the organism is isolated, then obtaining the centroid of the organism. The output is a time series of (*x*,*y*) position with the starting point set to (0,0), as well as the size (pixels) and perimeter at each frame. The organism has a tendency to climb walls and become difficult to segment once they arrive at the wall of the chamber, so the centroid trajectories generated would end when organisms reach the wall. One caveat is that because trajectories are centroid trajectories, there can be some recorded displacements that are less related to motion along the trajectory and more related to organism shape changes [38]. To mitigate the effects of the latter, for the windrose plots in figure 2*c,d*, we filtered out data points where the organism centroid moved equal to or less than 0.0601 mm (resolution limit) in both *x* and *y*.

### 2.3. Orientation experiment

Similar to other behaviour experiments, *T. adhaerens* were placed in nutrient-free ASW for 2–8 h prior to behavioural experiments in order for them to shed their undigested algae coat so as to remove chemical information from algae to isolate the effect of thermal information.

For the orientation experiment, we used a Squid tracking microscope which was developed in our laboratory and described by Li *et al*. [39]. The organism was placed in a circular glass-bottom chamber (WillCo Wells, GWST-5040). Polystyrene ‘sticky microspheres’ were prepared according to a procedure described by Prakash *et al*. [40] Briefly, PolyLink Protein Coupling Kit (for COOH microparticles, Polysciences, catalogue number 24350-1) was used to coat COOH-functionalized microspheres (31.7 µm diameter, from Spherotech CM-300-10) with wheat germ agglutinin (WGA; Molecular Probes, W11262). Then, the ‘sticky microspheres’ were sprinkled onto the organisms using a micropipette. The organism was tracked under the tracking microscope until the stage reached its limit.

The outputs of the tracking program include a TIFF stack (acquired at 0.5 frames s^−1^) and the stage coordinates. We manually tracked the positions of two beads over the course of the experiment. Using Fiji, we determined the angle *θ* as labelled in figure 2*f* as the angle between the line drawn from bead 1 to bead 2 and the positive *x*-axis.

### 2.4. Quantitative metrics

Evident in the raw trajectories in figure 1, the behavioural phenotypes that we observe show a lot of complexity across timescales. We thus chose to characterize the data by a myriad of metrics described below. Increasing the number of dimensions of each trajectory in the space of quantitative descriptors allows us to have a better toolkit that we can now use to explore possible mechanisms behind the phenotype by introducing perturbations into the assay that we have developed.

As measures of directedness of motion, we first considered mean velocities relative to thermal gradient axis. Mean velocities parallel and orthogonal to thermal gradient axis are determined as mean ‘momentary velocities' across sliding window length of *n* frames projected onto the thermal axis (or orthogonal axis), and the average is across non-overlapping intervals (unless specified—for some metrics where we used bigger window sizes, we opted for overlapping windows). In our analysis, we used non-overlapping sliding windows with *n* = 2 frames, and *δt* = *n*(30 s/frame) = 1 min unless otherwise indicated. Statistical tests were done using Scipy with Mann–Whitney *U*-rank test.

$$\left\langle v_{\mathrm{parallel}}(n)\right\rangle =\frac{1}{N_{I}}\overset{N_{I}}{\underset{i=1}{\sum }}\frac{\left[r(in)-r(in-n)\right]}{\delta t}\cos\;\theta ,$$

and

$$\left\langle v_{\mathrm{orthogonal}}(n)\right\rangle =\frac{1}{N_{I}}\overset{N_{I}}{\underset{i=1}{\sum }}\frac{\left[r(in)-r(in-n)\right]}{\delta t}\sin\;\theta .$$

Mean squared displacement (MSD) is a common way to characterize the trajectories. We computed MSD using window size of 1 (30 s intervals—highest temporal resolution available for our trajectories). Because we are interested in characterizing the directedness of motion along the thermal axis, we separated the MSD calculations into MSD parallel to the gradient axis (MSD_parallel_) and MSD orthogonal to the thermal axis (MSD_orthogonal_). The slope of the log–log MSD plot, also referred to as the *α* value, is a simple measure reflecting directional persistence; its value is 1 for random motion and 2 for a straight (ballistic) motion [38]. *α <* 1 corresponds to subdiffusive motion, and 1 *< α <* 2 corresponds to superdiffusive motion [41].

The straightness index [42] is a simple ratio of end-to-end displacement (*D*) to total distance travelled (*L*) [43].

$$M_{\mathrm{Straightness}}=\frac{D}{L}.$$

It is a good metric for quantifying the straightness of the trajectory but like MSD, is blind to direction. The directionality index is a slight modification of the straightness index to get an idea of efficiency of moving towards the thermal stimulus.

$$M_{\mathrm{Directionality}}=\frac{D\cos\theta }{L}.$$

Path persistence length was determined as described in [44] using the equation

$$\left\langle R^{2}\right\rangle =2L_{p}^{2}\left(\frac{L}{L_{p}}-1+e^{-L/L_{p}}\right).$$

For incremental values of *L* (window length—time is not fixed here, only the total distance travelled in each window) up to 2 cm, 〈*R*^2^〉 was calculated as the averaged squared displacement between non-overlapping contours of length *L*. Then, the parameter *L_p_* was determined using a least regression fit in MATLAB. Then, plotting *L* against *L_p_*, we determined the window length *L* for which we get the longest persistence length.

Another way to characterize persistence in movement direction is to fit the root-mean-squared displacement (RMSD) curves using Taylor's equation [45]

$$\mathrm{RMSD}(t)={\left[2v^{2}\tau \left(t-\tau \left(1-\exp\left(-\frac{t}{\tau }\right)\right)\right)\right]}^{1/2},$$

where the fit parameter *v* (mm min^−1^) is ballistic velocity and *τ* (min) is the decorrelation timescale of the direction of movement.

For trials with thermal gradient, we noted that for most trials, the RMSD plot can actually be better fit with

**Figure 3.**
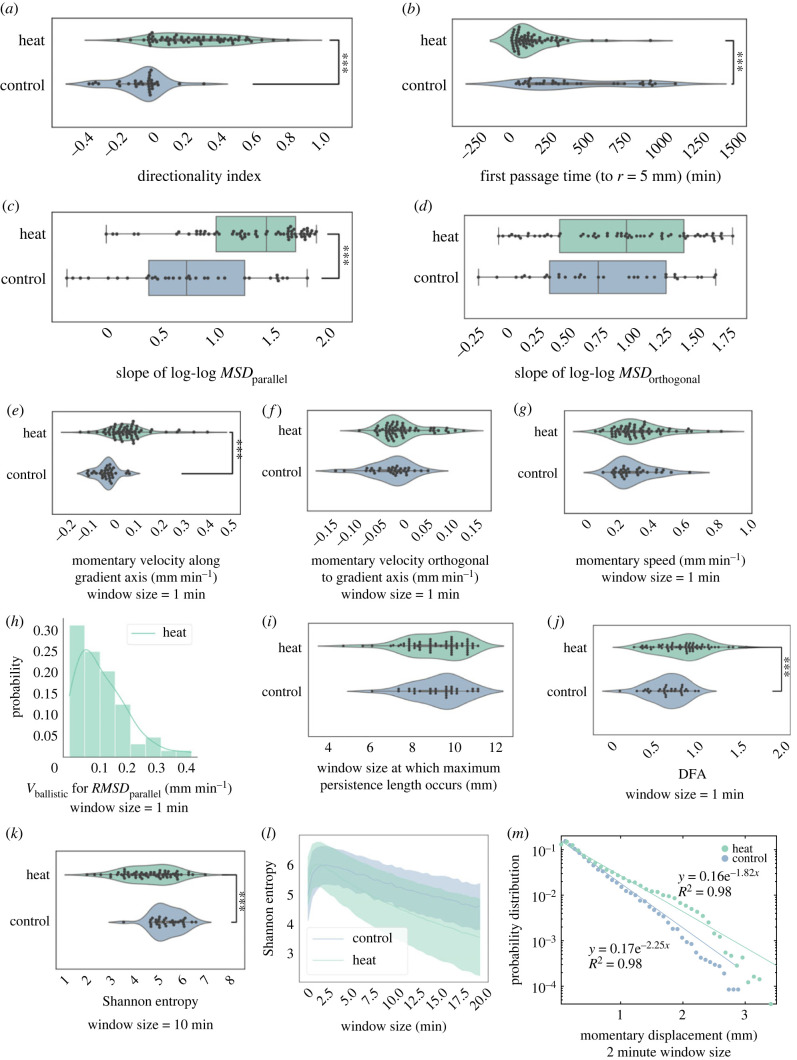
Various metrics to quantify directed motility in *T. adhaerens* undergoing thermotaxis. (*a*) With thermal gradient applied, we observe a significant increase in directionality index (overall displacement parallel to thermal gradient divided by total distance travelled). (*b*) We observe a significant decrease in first passage time (time for the organism to reach a radial distance of 5 mm from its starting point) with application of thermal gradient. (*c*) Slope of log–log MSD parallel to thermal gradient indicates increase in directedness of motion. Mean *±* s.e.: 1.33 *±* 0.06 for trials with thermal gradient, and 0.75 *±* 0.10 for trials without. (*d*) Slope of log–log MSD orthogonal to gradient remains unaffected. Mean *±* s.e.: 0.95 *±* 0.07 for trials with thermal gradient, and 0.82 *±* 0.09 for trials without. (*e*) Once the organism starts exploring, we observe an increase in mean momentary velocity parallel to the thermal gradient. (*f*) Mean momentary velocity orthogonal to gradient remains unaffected. (*g*) There is also no significant increase in mean momentary speed. (*h*) Once the organism starts exploring, the RMSD for trials under thermal gradient can mostly be fit using the linear ballistic motion estimate. (*i*) There is no significant difference between the window sizes that result in maximum persistence length for trials with and without thermal gradient. (*j*) Detrended fluctuation analysis shows a significant increase in long-range correlation in presence of thermal gradient. (*k*,*l*) Lower entropy seen with increasing sliding window size indicates increase in long-range correlation as a result of thermal gradient. (*m*) Step length distributions along gradient axis (for overlapping window size = 2 min) can be well fitted by single exponential functions. *p *<* 0.05, ***p *<* 0.001.

RMSD(t)=vt,

which is for ballistic motion. This is what we did for figure 3*h*.

For MSD and RMSD calculations, in order to normalize for the trajectory length, and to avoid any artefacts that may be associated with stagnant periods near the beginning (e.g. some organisms take some time to unfold) and end (e.g. some organisms touch the edge of the arena) of some trials, we computed the MSD along thermal gradient axis using the 200 time points between (last frame-200-10, last frame-10), which corresponds to a 100 min time window near the end of the trajectory, ending 5 min before the end of each trial.

Complexity metrics such as detrended fluctuation analysis (DFA) and Shannon entropy were computed using Neurokit [46]. DFA was originally introduced as a long-term memory metric that is able to differentiate local patchiness (mosaic structure of DNA where there are lots of local excess of a particular base pair) from long-range correlations [47]. This is useful for our data because we also see ‘local patchiness’ in momentary velocities, as the trajectories are ‘jittery’, with segments where the centroid of the organism is mostly stationary.

We tried fitting our step length histogram data (number of bins set to 40) with general power law, Lévy distribution $\left(f\;(x)={\left(c/2\pi \right)}^{1/2}(\exp(-c/(2(x-\mu )))/({\left(x-\mu \right)}^{3/2}))\right)$ and exponential function. The code for these fitting attempts can be found in our GitHub repository. The exponential fit was determined to be the better fit (*r*^2^-value = 0.97 as opposed to 0.9).

### 2.5. Identification of potentially relevant transient receptor potential channel homologues in *Trichoplax adhaerens*

We sought to ask whether TRP channel homologues are present in *T. adhaerens* and whether they may be relevant to the thermotaxis behaviour we observe. So, we start by searching for various TRP channels/homologues that are reported to be temperature-sensitive in other organisms. Then, we use NCBI BlastP to do reciprocal BLAST starting with accession numbers for those examples of TRP channels reported to be temperature-sensitive in other organisms. We include the top protein sequence pairs (in *T. adhaerens* and in the organism that we started with) that we found through reciprocal BLAST in the homology tree in figure 5. See our GitHub repository for detailed results of the reciprocal BlastP process, as well for a list of references where we found the starting sequences in various organisms.

Then, we used MUSCLE Alignment on Geneious software (maximum number of iterations set to eight) to perform multiple alignment, and again using Geneious, we constructed the homology tree in figure 5 using Geneious Tree Builder with default settings (Genetic distance model set to Jukes-Cantor, Tree building method set to Neighbor-joining, Outgroup set to No Outgroup).

### 2.6. Drug perturbation assays

Drug perturbation assays are done in the same chamber type as behavioural assays (5.25 cm ID PVC tubing on silicon wafer with flexible heating strip at bottom of wafer), and organisms are similarly placed inside nutrient-free environment and allowed to shed its algae prior to experiment. Naringenin (TCI chemicals, product number N0072) was dissolved in DMSO (Sigma-Aldrich, CAS number 67-68-5) to make a 10 mM stock solution. A new stock solution is made fresh on the day of each experiment. This stock solution is then diluted in ASW to a 500 ml solution immediately prior to addition, so that when added to the 25 ml chamber, the final naringenin concentration would be 40 µM (we did a concentration screen and picked the lowest concentration at which we saw a behavioural effect). Other drugs we tried but the results for which are not included in this manuscript include Flufenamic acid (Sigma-Aldrich, CAS number 530-78-9—anecdotally, inhibited thermotaxis at 10 µM but also inhibited motility in the limited number of trials we attempted), Ruthenium red (had no discernible effect) and U73122 (killed animals in all concentrations we tried).

## 3. Results

### 3.1. *Trichoplax adhaerens* demonstrates robust positive thermotaxis

To probe how *T. adhaerens* reacts to thermal gradients, we set up a 5.25 cm diameter (approx. 3 cm deep) experimental chamber with silicon wafer floor biased on one side with a polyimide flexible heater (figure 1*c*). Two independent methods were used for measurement of temperature gradients—thermistors lining the bottom of the silicon wafer for some chambers and an overhead infrared camera-based set-up for chambers without thermistors. We range our assay from 13.3°C to 28.2°C (with most trials falling in the range of 17°C to 22.5°C).

As controls, and to ensure parameters other than the thermal gradient do not influence our assay, we performed the experiments at various times of day to rule out the effect of potential circadian cycles (electronic supplementary material, figure S1*a*). Prior to running any experiments, organisms are transferred from the dishes with algae mat on which they are grown into nutrient-free dishes with only ASW for a few hours until they shed their undigested algae coat to ascertain that food stimuli and associated gradients are not present in the dish. Experiments are conducted sequentially with a single organism in the chamber at a time to eliminate potential effects of inter-organism communication. To avoid any influence of light, all experiments are conducted in the dark with only diffused overhead uniform illumination—just enough to spot the animal. See electronic supplementary material for further details on controls experiments.

When thermal gradient is applied, *T. adhaerens* displays directed motion towards the warmer side of the arena (figure 1*d*,*e*; electronic supplementary material, video S1). In contrast, without an applied thermal gradient (figure 1*f*; electronic supplementary material, video S1), in a similar timeframe, the organism tarries near its starting point. We repeated the thermal gradient and control assays 61 and 33 times, respectively. Comparing the motility tracks between these two conditions, it is evident that thermotaxis in *T. adhaerens* is a highly robust phenomenon (figure 1*g–h*). The application of a thermal gradient generates a very small surface flow of approximately 0.03 mm s^−1^ (determined by tracking a floating debris). As an additional control to ensure that the observed behaviour is not a result of this concomitant flow, we introduce a much stronger, externally generated, 3 mm s^−1^ confounding flow using a stir bar. We show that the organism is still able to find the thermal gradient (figure 2*g*; electronic supplementary material, figure S1b, video S6), demonstrating that this phenomenon is unperturbed by these flows.

Next, we look more closely at thermotaxis motility trajectories using a range of relevant parameters. Under thermal gradients ranging from 0.008 to 0.42°C cm^−1^, within the first 600 min, most organisms already show directed motion towards the thermal source (in the *y*-direction) (figure 2*a*). By contrast, in the control condition, within the first 600 min, organisms tend to stay near the starting point or venture in random directions (figure 2*b*). The distribution of momentary velocities for each condition reinforces the robustness of the phenomenon. When a thermal gradient is applied (figure 2*c*), motion is clearly more directed towards the thermal gradient. This is in contrast with the even distribution of directions in the control condition (figure 2*d*). When we introduce a sudden switch in gradient direction once the organism has already started moving up a thermal gradient, we observed (with the caveat that this is an anecdotal, single-trial observation) that it takes the organism approximately 20 min to successfully reverse directions (electronic supplementary material, video S4, figure 2*e*).

### 3.2. *Trichoplax adhaerens* does not maintain a fixed orientation in the animal's reference frame while performing thermotaxis

Since all extant animals other than *Placozoa* break body axis symmetry via establishment of AP symmetry, and inspired by the observation when watching raw videos (see electronic supplementary material, video S1, for example, and also figure 1*d*,*e* where we showed outlines of the organism at various time points) that the organism seems to constantly change shape and orientation, we explicitly ask how the orientation of *Placozoa*—an apolar animal—changes during thermotaxis. To ask whether the organism polarizes to perform thermotaxis or remains apolar with varying orientation in the animal's reference frame, we attach two WGA-coated polystyrene micro-beads (31.7 µm diameter) to the organism. We observe its thermotaxis behaviour under higher magnification via tracking microscopy [39] while also tracking the angle between the line segment connecting the two beads and the positive *x*-axis (figure 2*f*; electronic supplementary material, video S7). Notably, we confirm, as we also see in lower resolution videos, that *T. adhaerens* is able to establish a stable direction of movement along the thermal axis without a stable orientation in the frame of reference to the animal body (figure 2*f*). This finding suggests a distinct set of processes must be at play in how thermosensory machinery is coupled to ‘ciliary flocking’ [36] in an ever-rotating frame of reference to enable this behaviour.

A high degree of complexity exists across various length- and time-scales in *T. adhaerens* motility trajectories. In order to appreciate information embedded in these trajectories, it is useful to review how *T. adhaerens* walks on surfaces via rapidly beating cilia on its ventral surface. This unique mode of motility was recently characterized via direct real-time imaging of all cilia in a living motile organism [34–36]. Timescale of single cilia beat is approximately 0.2 s, and timescale for locomotive dynamics of the organism is approximately 10s of seconds [35]. Furthermore, something unique to *T. adhaerens* is the fact that its shape changes drastically while moving (electronic supplementary material, video S7, figure 1*d*,*e*). We recently established that the principles of *T. adhaerens* motility are embedded in the emergent phenomena of ciliary flocking dynamics [34–36]. Interspersed in these trajectories, the organism also demonstrates short timescale events such as freezing (approx. minutes), vortex insertion and vortex ejection (which leads to transition from rotation to translation motion). These shorter timescale events then cascade up to the long timescale taxis which we observe, linking a vast range of length- and time-scales. In the future, we intend to collect cellular resolution data of organisms navigating complex thermal environments, while simultaneously tracking ciliary activity at single cilia resolution. These datasets will link sensory and motor activity and further clarify how environmental information is read and processed by the current system.

### 3.3. Thermal gradient results in superdiffusive motion and increased long timescale correlation

Our thermotaxis assay gives us an organism-scale readout that embodies behavioural complexity across time- and size-scales. We next sought to quantitatively characterize these trajectories to find relevant metrics and timescales of the organism's response to thermal gradients. On the longest timescale observable from our experiments, which is the ‘end-to-end trajectory’ timescale, the application of a thermal gradient manifests in a significant (*p* *<* 0.0001) increase in directionality index (figure 3*a*). This metric is also referred to sometimes as thermotaxis efficiency index [33]. We also observe that with the application of a thermal gradient, overall trajectories are more aligned with the thermal gradient (figure 2*h*), and the organism spends more time in areas of the arena that are closer to the heat source than their starting point (figure 2*g*). On the shortest timescale of observation, we see a significant increase (*p* *<* 0.0001) in mean momentary velocity parallel to the thermal gradient (figure 3*e*). We confirm that this increase is not due to an increase in mean momentary speed (figure 3*g*), and it does not come with a concomitant increase in mean momentary velocity orthogonal to the thermal gradient (figure 3*f*).

We have previously established that timescales of directional persistence are dictated by ciliary flocking dynamics, and in particular by the introduction and ejection of ciliary vortices [36]. Here, we explore how the presence of a thermal gradient might affect persistence length in the organism trajectory. Looking at path persistence length [44], we find that window lengths of 7–11 mm result in maximum persistence length, and there is no significant change with application of thermal gradient (figure 3*i*). This suggests that the presence of a thermal gradient does not change inherent persistence patterns in organism-scale motion.

Even though the inherent persistence lengths in movement do not seem to be affected by the thermal gradient, the presence of the thermal gradient does increase long timescale correlation. With an applied thermal gradient, we find the organism demonstrates superdiffusive motion parallel to the gradient axis when thermal gradient is applied without a concomitant change in the component of motion orthogonal to the gradient axis (figure 3*c*,*d*). Under a thermal gradient, the root-mean-squared displacement parallel to the gradient axis can be well described by ballistic motion estimate (figure 3*h*). The increase in long-range correlation with application of thermal gradient also manifests in significant differences between thermal gradient and control conditions in DFA, and significant decrease in entropy as we increase sliding window size (figure 3*j*–*l*). Thus, even though in both conditions we see ‘segments’ of directed motion, the segments are more directionally aligned when a thermal gradient is applied.

### 3.4. Momentary displacement distribution is well described by exponential functions for both thermotaxis and control conditions

The observation of segments of directed motion, along with the significant decrease in first passage time (figure 3*b*) led us to ask whether sensory information induces new patterns of motion or organizes existing ones. We find that the distributions of momentary displacement for both organisms under thermal gradients and for control organisms can be well described by single exponential functions (figure 3*m*). This corresponds well with our previous work on *T. adhaerens* motility behaviour in zero-information environments—where flocking and cilia walking encodes a dynamical system that can be perturbed by various environmental parameters [34–36]. When introduced in a thermal gradient, this dynamical response is adapted, leading to *T. adhaerens* thermotaxis as a ‘motor-sensory’ response, wherein the thermal gradient is potentially modulating the longer timescale of directional drift of existing underlying patterns of motion [36,37].

### 3.5. *Trichoplax adhaerens* does not show preference for culture temperature

**Figure 4.**
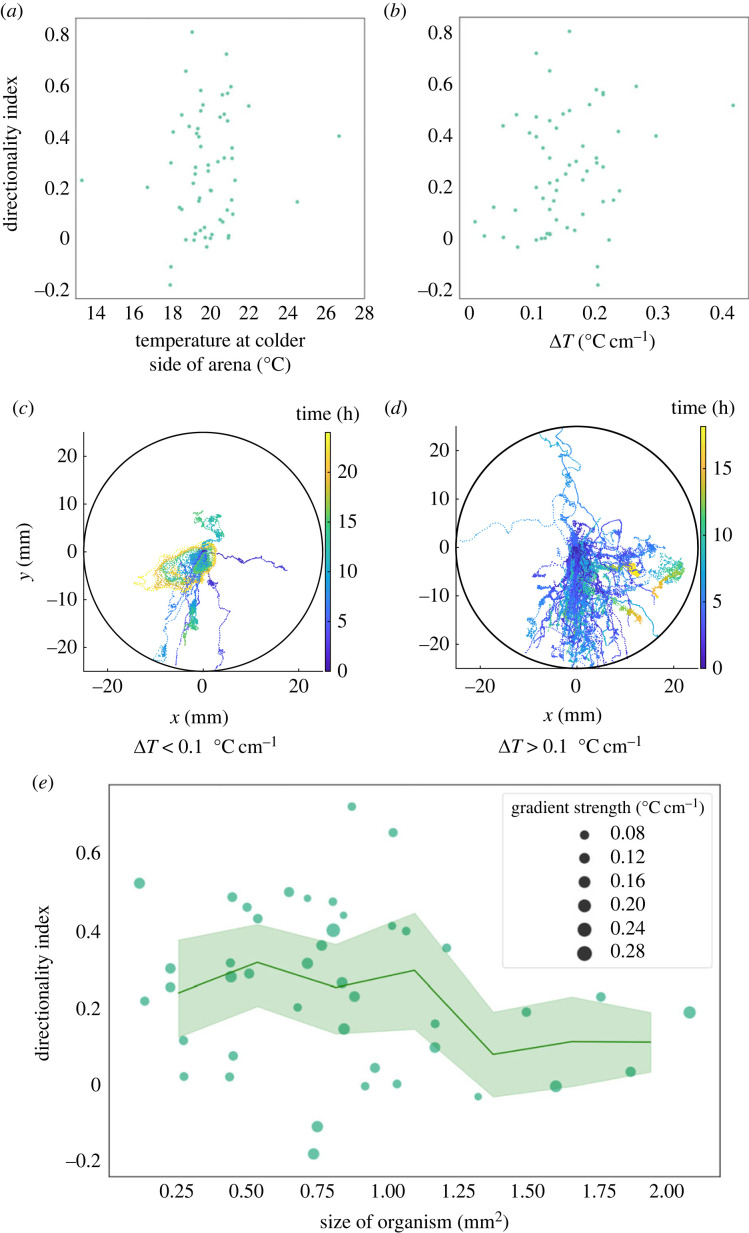
*Trichoplax adhaerens* can detect a thermal gradient of at least 0.1°C cm^−1^, and thermotaxis behaviour does not appear to be size-dependent. (*a*) There is no apparent correlation between temperature at the colder side of the arena (T2) and directionality index. (*b*) There does not appear to be a correlation between gradient strength and directionality index. (*c*,*d*) Binning trajectories by whether the gradient strength is below (*c*) or above (*d*) 0.1°C cm^−1^, we qualitatively see that even at low gradient strengths, the organism is mostly able to detect the gradient (see electronic supplementary material, figure S4 for the trajectories for trials with the three lowest Δ*T* that we tried; see electronic supplementary material, video S2 for video of trial with shallowest gradient). (*e*) We do not observe any clear dependencies of directionality index on size of organism (note that *n* = 43 as opposed to 61 in this figure because we did not succeed in measuring size of all organisms).

In addition to search strategy, thermotaxis behaviour is also interesting in the context of ecology, adaptation and size-scaling. So, next we ask how parameters such as absolute temperature, gradient strength and size of organism affect thermotaxis behaviour. We explore if, like for other organisms such as *C. elegans*, *T. adhaerens* thermotaxis shows a preference for culture temperature. We test T2 (temperature on colder side of the arena) ranging from 13.3°C to 26.7°C, which spans both sides of our culture temperature (18°C), with the T2 for the bulk of the trials falling between 17°C and 22.5°C. *Trichoplax adhaerens* does not appear to show a preference towards its culture temperature—in the bulk of our trials, the temperatures are above culture temperature and the organism performs positive thermotaxis (i.e. towards the warmer side of the arena and further away from culture temperature). Within the limited range of temperatures tested, there are no absolute-temperature dependent trends in directionality index (figure 4*a*), and the organism shows robust positive thermotaxis through this entire temperature range.

### 3.6. *Trichoplax adhaerens* can detect gradients of at least of 0.1°C cm^−1^

We sought to determine whether the strength of the thermal gradient affects *T. adhaerens* thermotaxis. We do not observe any apparent dependence of directionality index on Δ*T* (figure 4*b*). Typically, a directionality index above 0 is considered positive thermotaxis. If we take 0 as the cut-off for positive thermotaxis, then 89% and 90% of organisms demonstrate positive thermotaxis at gradients below and above 0.1°C cm^−1^, respectively. If we define ‘effective thermotaxis' as thermotaxis with a directionality index higher than 0.1, then 56% of organisms in our experiments effectively thermotax at gradient strengths below 0.1°C cm^−1^ (figure 4*b*,*c*). At gradient strengths above 0.1°C cm^−1^, 75% of organisms effectively thermotax (figure 4*b*,*d*). Thus, we can safely conclude that the organism is able to detect temperature gradients of at least 0.1°C cm^−1^. The lowest temperature gradient that *T. adhaerens* is able to detect in our experiments was 0.008°C cm^−1^ (electronic supplementary material, video S2, figure S4).

### 3.7. There is no apparent size dependency of directionality index in *Trichoplax adhaerens* thermotaxis

*Trichoplax adhaerens* naturally shows orders of magnitude variations in size. To probe the effect of size on thermotaxis behaviour, we select naturally occurring organisms of various sizes. In our experiments, the smallest organism measures 0.12 mm^2^ while the largest organism is 2.1 mm^2^ in size. Roughly estimating the area of each cell to be 30 µm^2^ and the organism as having two cell layers [48], this translates to a size range of less than 10 000 cells to approximately 135 000 cells.

We do not find direct dependencies of directionality index on size (figure 4*e*). If *T. adhaerens* has a scale-free capacity for information integration without degradation in performance as the size of the organism increases, it provides us new avenues to explore how such a scale-free algorithm for gradient detection might be implemented in the organism. How organisms can sense such shallow gradients and why there appears to be no size dependency also leads us to ask what the thermosensory circuitry looks like. To get a handle on the circuitry, we ask what thermosensitive ion channels might be present in *T. adhaerens*.

### 3.8. Transient receptor potential channel homologues may be temperature sensors in *Trichoplax adhaerens*

In seeking to identify potential sensors mediating the thermotaxis response, we start by looking to TRP channels. TRP channels have diverse known functions; they act as ubiquitous sensors in vertebrates and invertebrates alike and are responsive to numerous chemical and physical stimuli, including temperature [49–59]. These channels are additionally interesting because they are often polymodally activated and are considered an example of signal integration at the level of the sensor itself [53,60]. TRP channel homologues have been identified from the *T. adhaerens* genome—specifically TRPM1-4, TRPML, TRPP1-2 and TRPV1-2 [61,62].

**Figure 5.**
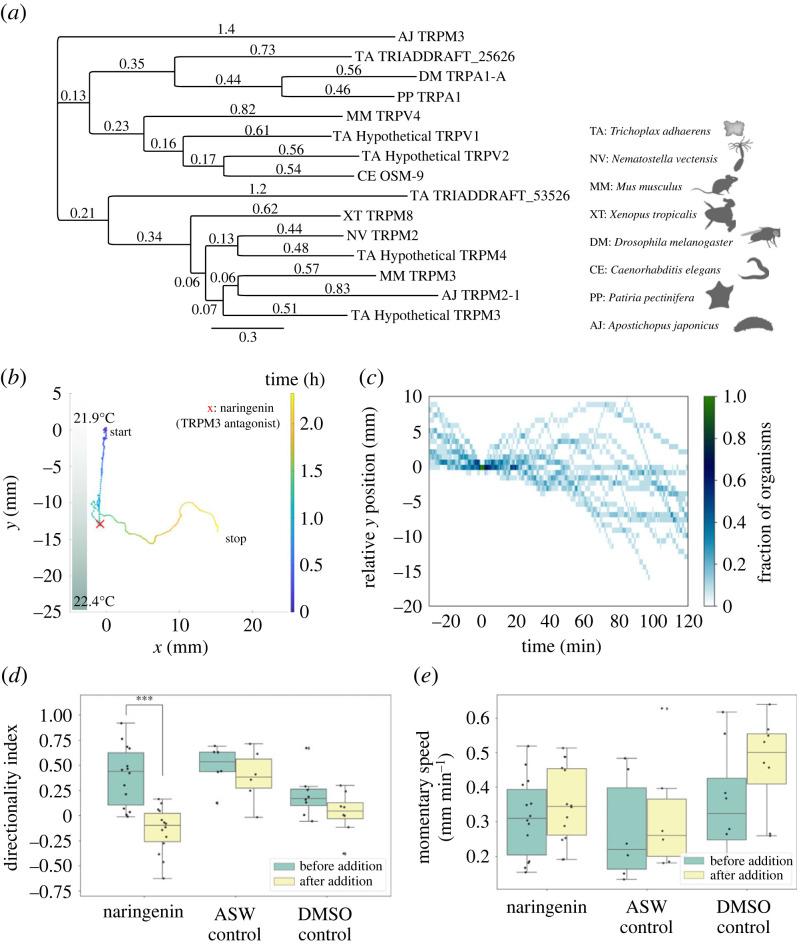
TRP channel homologues are potentially involved in signal cascade driving response to thermal stimulus. (*a*) Homology tree incorporating TRP channels with reported temperature-sensitivity in other organisms across the metazoan tree of life, and their top reciprocal best hits in *T. adhaerens*. Accession numbers for all proteins shown in the tree are found in electronic supplementary material, table S1. From this, we broadly see three groups of *T. adhaerens* hypothetical TRP channels, with two top hits not having previously been identified as hypothetical TRP channels. (*b*) A sample trajectory under the indicated thermal gradient, with 40 µM naringenin (known TRPM3 antagonist) added at the time point indicated by the red cross symbols. Naringenin addition inhibits thermotaxis behaviour, but the organism seems to recover after approximately an hour. (*c*) Kymograph showing positions along thermal axis relative to the position at naringenin addition for *n* = 14. Naringenin robustly inhibits thermotaxis, and the inhibition appears to be transient, with most organisms recovering after an hour. (*d,e*) Boxplots showing directionality index along gradient axis (*d*) and mean momentary speed (*e*) in 20 min time windows before and after addition of naringenin (TRPM3 antagonist). Sliding window size is 30 s, non-overlapping windows. It appears naringenin inhibits thermotaxis (*d*), and the observed thermotaxis inhibition is not due to the DMSO solvent used nor the seawater that is used for dilution (more details on controls in electronic supplementary material, figure S1*c,d*). Naringenin inhibits thermotaxis without reducing motility (*e*). *p *<* 0.05, ***p *<* 0.001.

Temperature responsiveness of TRP channels has been identified across a wide range of organisms. Some examples of TRP channels that have been shown to be temperature-sensitive are TRPM2-1 in *Apostichopus japonicus* [54], OSM-9 and OCR-2 TRPV channels in *C. elegans* [55], TRPA1 channels in *Drosophila melanogaster* [56], TRPM8 channels in *Xenopus tropicalis* [57], TRPM2 channels in *Nematostella vectensis*, and channels such as TRPV4 and TRPM3 in *Mus musculus* [59,63,64]. We found reciprocal best hits in *T. adhaerens* using blastp and starting with each of these channels, and from the homology tree that we were able to construct using the reciprocal best hits (figure 5*a*), we are able to see three main groups of hypothetical TRP channels in *T. adhaerens*. Interesting to note that this is a subset of the hypothetical TRP channel homologues identified previously, and reciprocal blastp returned two additional hypothetical proteins (accession numbers XP_002109464 and XP_002112601) that were not previously identified as hypothetical TRP channel homologues. From these top hits, *T. adhaerens* hypothetical TRPM3 and TRPM4 were our starting places of interest because they showed up multiple times as reciprocal blastp top hits, and because a specific inhibitor, naringenin [64,65], has been identified for *Mus musculus* TRPM3 channel, which is close in homology to *T. adhaerens* hypothetical TRPM3 (figure 5*a*).

We find that thermotaxis behaviour is interrupted by addition of 40 µM naringenin (figure 5*b*; electronic supplementary material, video S3). This inhibition of thermotaxis by naringenin is robust across 14 biologically independent trials (figure 5*c*). The inhibition is temporary and reversible: most organisms recover positive thermotaxis behaviour within one hour post-naringenin addition or following washout (figure 5*b*; electronic supplementary material, figure S1*d*). The inhibition also occurs without concomitantly inhibiting organism motility (figure 5*d*,*e*). The observed inhibition of thermotaxis by naringenin, which occurs without motility inhibition suggests that a TRP channel homologue (perhaps a TRPM3 channel homologue) may be a temperature sensor in *T. adhaerens*. However, this suggestion has the caveat that naringenin may have other effects that we are not aware of on the organism [66], which will be further explored in future work.

## 4. Discussion

All benthic organisms face a complex seascape of environmental conditions. Many organisms such as slime moulds and *C. elegans* show preference for their culture temperature, and their thermotaxis behaviour can be altered by changing culture conditions. By running a range of experiments at various baseline temperatures, we find *T. adhaerens* thermotaxis does not exhibit a preference for culture temperature (18°C). Since *T. adhaerens* behaviour has never been observed in its native environment, our work establishes an environmental parameter that elicits a robust response in the organism. In context of benthic ecosystems, decision making might rely on a broad range of signals such as light, salinity etc., instead of one single preferred temperature. In the future, we intend to explore multi-sensory stimuli in our arenas. Decision making in a multi-sensory stimuli arena might also benefit from the fact that *T. adhaerens* does not break symmetry and remains apolar in its frame of reference.

Ciliary dynamics play a key role in understanding how *T. adhaerens* performs thermotaxis as an unpolarized tissue. Here we have an organism that does not have an inherent AP axis—hence ideas like heading do not really apply, and the ‘momentary displacements' we show in figure 3*m* does not necessarily correlate to ‘oriented runs’. Rather, the motion and directedness are cascaded up from and governed by ciliary flocking dynamics, where coherent, directed motion is produced as a result of cell–cell interactions [35]. This corresponds well with our data in figure 3 that suggest the thermal gradient stimulus serves to modulate intrinsic patterns of motion generated by ciliary flocking dynamics that exist even in zero-information environments. The discovery of thermotaxis in *T. adhaerens* as a behavioural handle thus opens up a host of interesting opportunities for insights that can be gained by simultaneously recording ciliary activity while the organism performs thermotaxis—what do larger ‘momentary displacements' look like in terms of ciliary dynamics? Does the organism encode memory somehow in ciliary dynamics? Perhaps not doing so would allow it to be more adaptive in multi-sensory and variable environments.

In many motor-sensory and sensory-motor systems, it is possible to ask what an optimal sensor-actuator ratio might be. Where might these sensors be located? Several models of integration of information along the sensory-motor axis are feasible in the frameworks of the discovery of thermotaxis described in *T. adhaerens*. Assuming that individual cilia themselves are sensory organelles, several models can be built for modulation of gliding motility as a function of thermal gradient [36]. Such a possibility has been previously shown for light-driven adhesion modulation in cilia in *Chlamydomonas* [67]. In this case, cilia as sensors will scale linearly with the size of the organism. Another possibility is existence of specific sensory cells that release particular peptides or small molecules involved in *T. adhaerens* behaviour modulation. We currently do not know where the temperature sensing cells might be located in the organism. Support for TRPM3's role in driving peptide or small molecule release has previously been shown [68,69]. Several neuropeptides that modulate *T. adhaerens* have already been demonstrated [70], and the recent work of Nikitin *et al*. presents a large amount of data on normal *T. adhaerens* locomotion and modulatory effects of amino acids transmitters and ATP [71]. In the future, we intend to explore how thermotaxis links to neuropeptide/transmitter-based behavioural control. In addition, it is also feasible that mucus trails observed previously [72] in *T. adhaerens* might be able to encode spatial memory and modulate thermotaxis.

Through our experiments, we have found that thermotaxis behaviour does not directly correlate with size of the organism. This finding is intriguing, as previously, optimal cluster size in collective migration has been seen both theoretically [73] and experimentally (in context of border cells in *Drosophila* egg chamber) [74]. Although these previous findings are relevant, significant differences exist in how *T. adhaerens* glides on surfaces due to ‘ciliary flocking’. Since the numbers of cells and cilia in an organism scale linearly with size, above a certain size, coherence in ciliary flocks may decrease with size [34], making it more difficult for larger tissues to change course. On the other hand, larger sizes may also potentially correspond to more sensors. Perhaps related to size, perimeter-to-area ratio may also be a relevant parameter—we observe that organisms with anomalous perimeter-to-area ratio struggle in performing thermotaxis (electronic supplementary material, video S5). Size may also be related to the shape repertoire available to an organism. These factors may all be rooted in ciliary flocking dynamics. Our findings lead to many interesting questions in the realm of collective migration—what is the circuitry behind this scale-free behavioural response that we observe? For this question, an exciting future direction would be to directly observe ciliary flocking dynamics when a thermal stimulus is presented.

Finally, since complex ciliary flocking dynamics such as vortex injection, ejection, merging and splitting are all involved in encoding motility behaviour in *T. adhaerens*, a higher order combination and control of these phenotypes might be involved in encoding thermotaxis. As we demonstrate in our experiments, organisms take up to hours to detect and respond to thermal cues. The vast gap in timescales between ciliary flocking dynamics and the time for detecting and climbing a gradient presents a fertile ground to explore a number of ideas in the space of motor modulation by sensory information.

Our current work in this non-model system—*T. adhaerens*—lays the foundation for studying higher order sensory and information processing capabilities of this seemingly simple animal. By building a rigorous experimental framework of tracking animals making decisions in complex artificial sensory seascapes, we believe a large number of questions pertaining to information processing in early multicellular life forms can be now formulated. For instance, how localized is the processing of information such as in reading a complex gradient?

## 5. Conclusion

In this work, we show the discovery of robust thermotaxis in *T. adhaerens*, a simple multicellular organism of the phylum *Placozoa* with no known neurons or AP symmetry breaking. We demonstrate a framework for conducting systematic, long-term behavioural assays for *T. adhaerens*. We characterize the trajectories using a myriad of metrics relating to directedness of motion and physics of behaviour, and find that the behavioural data corresponds with what we know about cilia flocking [34–36] to suggest that the thermal gradient stimulus modulates motion generated by ciliary flocking dynamics that exist even in zero-information environments. Further, we find that *T. adhaerens* can detect thermal gradients of at least 0.1°C cm^−1^. Notably, we show that unlike many other organisms that perform thermotaxis, *T. adhaerens* does not show a temperature preference that is related to culture conditions. It also does not maintain a fixed orientation in the frame of reference of its body while performing thermotaxis. We further report the apparent lack of dependence on size of the thermotaxis behaviour. Combined with our finding that TRP channel homologues might be potential temperature sensors in this system, we establish thermotaxis in *T. adhaerens* as a tractable behavioural handle to ask fascinating questions surrounding how an apolar tissue can respond to sensory signals through distributed signal processing. The rigorous behaviour assay we describe and our findings create a path to explore decision-making mechanisms that control both short- and long-term behaviour in this non-model, multicellular system at the ‘no brain limit’.

The question of different types of strategies in responding to thermal cues and what they could mean in an ecological context could also take on a particular importance in the context of climate change—how might changing climate affect behavioural response, and what impact could it have on organism survival? This question carries a sombre significance for an organism where we have only discovered a handful of known species (less than 10—all found in tropical waters) associated with the entire phyla.

## Data Availability

Descriptions of trial conditions, all centroid trajectories, analysis codes, additional analyses that did not make it into the papers, etc., are available from the GitHub repository: https://github.com/prakashlab/Trichoplax_Thermotaxis [75]. The data are provided in electronic supplementary material [76].
